# Gut microbial diversity in health and disease: experience of healthy Indian subjects, and colon carcinoma and inflammatory bowel disease patients

**DOI:** 10.1080/16512235.2017.1322447

**Published:** 2017-05-19

**Authors:** V. Deepak Bamola, Arnab Ghosh, Raj Kishor Kapardar, Banwari Lal, Simrita Cheema, Priyangshu Sarma, Rama Chaudhry

**Affiliations:** ^a^ Department of Microbiology, All India Institute of Medical Sciences, New Delhi, India; ^b^ Microbial Biotechnology Division, The Energy and Resources Institute, New Delhi, India

**Keywords:** Indian vegetarian, colon cancer, IBD, gut microbiota, India

## Abstract

**Background**: The intestinal microbiota, through complex interactions with the gut mucosa, play a key role in the pathogenesis of colon carcinoma and inflammatory bowel disease (IBD). The disease condition and dietary habits both influence gut microbial diversity.

**Objective**: The aim of this study was to assess the gut microbial profile of healthy subjects and patients with colon carcinoma and IBD. Healthy subjects included ‘Indian vegetarians/lactovegetarians’, who eat plant produce, milk and milk products, and ‘Indian non-vegetarians’, who eat plant produce, milk and milk products, certain meats and fish, and the eggs of certain birds and fish. ‘Indian vegetarians’ are different from ‘vegans’, who do not eat any foods derived wholly or partly from animals, including milk products.

**Design**: Stool samples were collected from healthy Indian vegetarians/lactovegetarians and non-vegetarians, and colon cancer and IBD patients. Clonal libraries of 16S ribosomal DNA (rDNA) of bacteria were created from each sample. Clones were sequenced from one representative sample of each group. Approximately 500 white colonies were picked at random from each sample and 100 colonies were sequenced after amplified rDNA restriction analysis.

**Results**: The dominant phylum from the healthy vegetarian was Firmicutes (34%), followed by Bacteroidetes (15%). The balance was reversed in the healthy non-vegetarian (Bacteroidetes 84%, Firmicutes 4%; ratio 21:1). The colon cancer and IBD patients had higher percentages of Bacteroidetes (55% in both) than Firmicutes (26% and 12%, respectively) but lower Bacteroidetes:Firmicutes ratios (3.8:1 and 2.4:1, respectively) than the healthy non-vegetarian. Bacterial phyla of Verrucomicrobiota and Actinobacteria were detected in 23% and 5% of IBD and colon patients, respectively.

**Conclusions**: Ribosomal Database Project profiling of gut flora in this study population showed remarkable differences, with unique diversity attributed to different diets and disease conditions.

## Introduction

The human gastrointestinal tract is one of the major surfaces for microbial colonization, with an estimated bacterial cell count of 10^11^–10^12^ per gram of content in the colon [3], representing approximately 70% of all microbes in the human body [4,5]. Interactions between the microbiota and the gut mucosa play an important role in human health and disease. Studies have revealed that the gut microbiome is responsible for the maturation and development of mucosal and systemic immunity, protection of the host against pathogens by production of antimicrobial substances, and maintenance of intestinal epithelial homeostasis and surface maturity [6–8]. Diverse metabolic processes of gut microbiota are involved in nutrient assimilation and xenobiotic processing in the host [9]. The influence of gut flora on obesity has been demonstrated in mouse models [10]. Imbalance in the composition of gut microbial communities is associated with the development of a number of gastrointestinal disorders, such as inflammatory bowel disease (IBD) and colon carcinoma [11,12].

Dysbiosis of the gut microbiota is associated with the pathogenesis of both intestinal disorders, including IBD, irritable bowel syndrome, and colon cancer, and extraintestinal disorders, including allergy, asthma, metabolic syndrome, and cardiovascular disease [13]. Changes in the microbial content of the gut also lead to an imbalance between the beneficial metabolic byproducts and bacterial toxic compounds [14]. Furthermore, both culture-dependent methods and molecular approaches have shown that dysbiosis of the gut bacterial community leads to overexpression of antigenic bacterial surface proteins, which results in an exaggerated immune response and chronic immune-mediated inflammatory mucosal damage in IBD patients [15–17]. In stools from patients with colorectal carcinoma, an increase in the proportion of bacteria such as *Bacteroides* spp. and *Prevotella* spp., along with an upsurge in the diversity of *Clostridium* spp., *Lactobacillus* spp., and *Eubacterium* spp., was observed compared to healthy cancer-free controls [18,19]. Although the role of an imbalance of gut microbiota in the development of colon carcinoma is still debatable, studies have suggested that altered flora may act as a major trigger for the initiation of tumorigenesis [20,21]. The colonic mucosa is thus being continuously influenced by the composition of commensal bacteria and their metabolites.

The main obstacle in characterization of the human gut flora is the lack of favorable conditions in laboratories for culturing this diverse group of bacteria, the majority of which are obligate anaerobes [22]. With the advent of high-throughput sequencing technologies, advances in bioinformatics, and the refinement of DNA amplification methods, robust analyses including phylogenetic and functional diversity of non-cultivable gut microbiota are now possible. Researchers around the globe have indicated the feasibility and applicability of such techniques for the gut microflora [23,24]. However, few studies have been carried out in the Indian population [25–28]. India has the second largest population in the world, comprising diverse ethnic and tribal groups, which are currently undergoing major cultural, socioeconomic, and technological transformations. The composition of the gut microbiota is also dependent on exogenous (e.g. diet) and endogenous (e.g. host genetics) variables, and varies from one geographical locale to another [3,^29^]. Since the dietary habits of the Indian population are different from those of the Western world, the findings of studies on colonic microbial diversity using the metagenomic approach in various Western populations, both healthy and diseased, cannot be extrapolated to their Indian counterparts.

In India, vegetarians/lactovegetarians eat plant produce, milk and milk products only, while non-vegetarians eat plant produce, milk and milk products, certain meats and fish, and the eggs of certain birds and fish [1] ‘Indian vegetarians’ are entirely different from ‘vegans’, who do not eat any foods derived wholly or partly from animals, including milk [2].

Information is almost completely lacking on the gut microbiota of the Indian population. Therefore, we have initiated a study to assess gut microbial diversity in the Indian population with reference to health status, dietary habits, and disease states. This study is designed to understand the gut microbiota and their metabolic end-products in healthy (vegetarian and non-vegetarian) adults and diseased (IBD and colon cancer) patients, and to understand the microbial diversity in the Indian population.

## Materials and methods

The study was conducted at the Department of Microbiology, All India Institute of Medical Sciences (AIIMS), New Delhi, India, in collaboration with The Energy Research Institute (TERI), New Delhi, India.

### Ethics and consent

Ethical clearance for the study was obtained by the Institutional Ethics Committee of AIIMS, New Delhi, India (ethical clearance reference numbers IEC/NP-365/2011 and RP-02/2012). Before enrollment, the study was explained to all participants and informed written consent to participate in the study was obtained from each subject.

### Subjects

Healthy adults included in the study were classified into two groups: subjects on an Indian vegetarian diet (who consume plant produce, milk and milk products) and those on a non-vegetarian diet (who eat eggs at least three or four times and meat/fish twice a week). Patients included in the study were also classified into two groups: patients with IBD and those with colon carcinoma. Exclusion criteria for all participants included a history of any probiotic intake in 2 weeks before sample collection and a history of antibiotic therapy within 2 months of study participation. Pregnant females and lactating mothers were also excluded from the study. Patients with IBD and colon carcinoma were included only when the diagnosis had been histopathologically confirmed and other comorbidities had been ruled out. Clinicodemographic data were collected from the patients in the form of a structured questionnaire at the time of sample collection.

### Isolation of fecal microbiota by conventional method

Eight stool samples were collected from each of the four groups (total 32 samples). Stool samples were collected in sterile wide-mouthed containers with tight-fitting lids.

Different bacterial culture media were used to isolate the aerobic and anaerobic bacteria from the stool samples. The media used were MacConkey agar, blood agar, bacteroides bile esculin agar (BBEA), brain–heart infusion blood agar (BHIBA), cycloserine–cefoxitin fructose agar (CCFA), and de Man–Rogosa–Sharpe agar (MRSA). MacConkey agar and blood agar were used to detect aerobic bacteria. BHIBA was used to detect anaerobic bacteria present in the sample and CCFA was used to detect *Clostridium difficile*. BBEA was used to detect *Bacteroides fragilis*. MRSA was used to detect *Lactobacillus* spp. To detect spore-bearing *Clostridia* spp., stool samples were subjected to alcohol shock treatment before culturing on BHIBA and CCFA, wherein about 0.5 ml of the sample was first treated with equal amounts of 100% ethanol for 60 min at 37°C (alcohol treatment) before plating. Metronidazole discs (5 µg) were then placed on to CCFA, BHIBA, and BBEA media. All the media except MacConkey agar were incubated anaerobically at 37°C in an anaerobic chamber for 48 h. The MacConkey plates were incubated aerobically at 37°C for 24 h. The isolated colonies were identified by conventional biochemical assays and Analytical Profile Index (API®; bioMérieux, Durham, NC, USA) API 20 A and API 50 CH.

### DNA isolation from fecal sample

Genomic DNA was isolated from human stool samples (wet weight 0.2 g) using the QIAmp DNA stool kit (Qiagen, Hilden, Germany), following the manufacturer’s instructions. Recovered DNA was quantified by spectrophotometric quantification (Nanodrop ND-2000 v.3.3.1; Nanodrop Technologies, Wilmington, DE, USA) and confirmed by electrophoresis on 1% agarose gel.

### 16S ribosomal DNA (rDNA) polymerase chain reaction (PCR) amplification and construction of clonal libraries

Amplification of 16S rDNA by PCR was performed in 25 μl of reaction mixture containing 2.5 mM/µl 10× PCR buffer [1× PCR buffer is 10 mmol/l Tris–HCl (pH 8.8 at 25°C), 50 mmol/l KCl, and 0.1% Triton X-100], 0.5 mM/µl MgCl_2_, 1.6 mM/µl deoxynucleoside triphosphate (dNTP), 0.2 μM/µl each of forward and reverse primers (27F-AGA GTT TGA TCC TGG CTC AG and 1492R-ACG GTT ACC TTG TTA CGA CTT, respectively), 0.1 units/µl of *Taq* DNA polymerase (NEB, Hitchin, UK), and 50 ng of template DNA. The PCR conditions used were an initial cycle of denaturation at 95°C for 5 min followed by 30 cycles of denaturation at 94°C for 1 min, annealing at 55°C for 1 min and extension at 72°C for 1 min 30 s, and a final extension step of 72°C for 10 min in a thermal cycler (Mastercycler® personal; Eppendorf, Hamburg, Germany). The PCR products were run on 1.5% agarose gel and visualized under an ultraviolet (UV) transilluminator. Amplified PCR products were purified using the QIA quick Gel Extraction Kit (Qiagen, Hilden, Germany). Purified products were subsequently ligated into pGEM®-T Easy Vector (Promega, Madison, WI, USA) and used to transform *Escherichia coli* DH5α cells (Promega) according to the manufacturer’s protocol. The positive clones were selected and stored in 96-well plates containing Luria broth freeze medium containing ampicillin (100 μg/ml).

### Construction of 16S rDNA clonal libraries

For each study participant, 96 clones from each stool sample were created. Plasmid inserts were PCRamplified by vector-specific primers, M13F (5´-GTAAAACGACGGCCA3´) and M13R (5´-AGGAAACAGCTATGAC3´). The PCR mixture consisted of 2.5 mM/µl 10× PCR buffer [1× PCR buffer is 10 mmol/l Tris–HCl (pH 8.8 at 25°C), 50 mmol/l KCl, and 0.1% Triton X-100], 2.5 mM MgCl_2_, 0.1 mM of each primer, 0.2 mM dNTP (Fermentas, Burlington Ontario, Canada), 1 U Taq polymerase (NEB, Hitchin, UK), and 50 ng of template DNA. The PCR cycling conditions included an initial denaturation step at 95°C for 5 min followed by 30 cycles of 94°C for 1 min, 55°C for 1 min, and 72°C for 1 min 30 s, and a final extension step of 72°C for 10 min. The presence of insert was confirmed by randomly picking 10 reactions and loading 5 μl of each of the amplified insert on a 1.5% agarose gel, followed by gel electrophoresis and visualization under a UV transilluminator. One subject from each study group was randomly selected and the amplified inserts of the clonal library of those study participants were subjected to sequencing. Approximately 500 white colonies were picked at random from each sample and 100 colonies were sequenced after amplified rDNA restriction analysis (ARDRA).

### ARDRA

Amplified inserts from the each of the libraries were selected for ARDRA. In brief, 1 μl of the lysed colony of the transformants was digested with HaeIII (20 U/μl) by incubation at 37°C for 2 h. The reaction was inactivated by adding 6× loading dye. The 96 digested clones from each library were then loaded on to 2% agarose gel stained with ethidium bromide and visualized in UV light. Depending on the difference in the number of band patterns for each of the samples, libraries were then selected for further sequencing.

Since sequencing of all the clonal libraries is expensive, a basic approach of clarifying the microbial communities using ARDRA was employed. ARDRA is a commonly used tool to study microbial diversity that relies on DNA polymorphism. HaeIII-ARDRA analysis proved very useful in the preliminary selection of samples belonging to the same group for sequencing. HaeIII-ARDRA has been performed specifically for gut microflora in many studies and therefore was chosen here. The unique patterns for the clonal libraries from different groups were determined. Based on the higher number of patterns, those samples were sequenced for the complete library.

### Sequencing and Ribosomal Database Project (RDP) analysis

The sequencing of the clonal libraries was then outsourced (Macrogen, Seoul, Republic of Korea). Bacteria were identified by performing Basic Local Alignment Search Tool (BLAST) analysis and selecting search results with at least 97% nucleotide similarity.

The taxonomic distinction between the data sets was revealed by comparing and mapping representative sequences using the RDP (https://rdp.cme.msu.edu/comparison/comp.jsp). This resulted in hierarchical mapping of the number of representative sequences at various taxonomic levels.

Since we did not have a large amount of data (96 sequences from each group), we manually selected or filtered the sequences from the raw data by looking at the chromatogram of the sequence. The Mallard program was used to check for chimera [30]. All poor-quality sequences and chimeras were removed before analysis.

### Statistical analysis

Data were reanalyzed using STATA version 11.2 software (StataCorp, College Station, TX, USA). For comparisons between different groups, one-way analysis of variance was applied, followed by Bonferroni correction. Where normality conditions were not fulfilled, the Kruskal–Wallis test was applied. A *p* value < 0.05 was considered statistically significant.

## Results

In this study, we have recruited a total of 32 subjects (eight subjects from different population subsets/groups, including healthy adults on a vegetarian diet, healthy adults on a non-vegetarian diet, patients with IBD, and patients with colon carcinoma). The mean age of the healthy vegetarians was 31.7 ± 5.9 years (range 26–44 years) with a male:female ratio of 5:3, while the mean age of the non-vegetarians was 34.1 ± 9.0 years (range 25–48 years) with a male:female ratio of 3:1. The mean body mass index (BMI) of healthy vegetarian and non-vegetarian adults was 23.2 ± 2.1 kg/m^2^ (range 19.9–25.23 kg/m^2^) and 24.2 ± 1.2 kg/m^2^ (range 24.91–26.33 kg/m^2^). There was no significant difference in BMI between these two groups (*p *= 0.263). The mean age of the IBD patients was 31.5 ± 12.6 years (range 22–44 years), and the mean age of the patients with colon carcinoma was 50.5 ± 12.2 years (range 42–70 years).

### Stool culture for gut microbiota

Different bacterial species were isolated using conventional culture methods. The most commonly isolated bacteria were *Escherichia coli*, *Citrobacter koseri*, *Enterococcus* spp., *Staphylococcus* spp., *Lactobacillus* spp., and *Clostridium* spp. *Escherichia coli* was isolated from all samples. *Lactobacilli* spp. were isolated from 62.5% (5/8) of healthy controls on a vegetarian diet and 50% (4/8) of healthy controls on a non-vegetarian diet. The culture positivity of lactobacilli was 12.5% (1/8) in IBD patients. No lactobacilli were isolated from patients with colon carcinoma. *Clostridium* spp. were found in 25% (2/8) patients with colon cancer, while no other group was positive for *Clostridium* spp.

### Microbial community structure

#### Construction of clonal libraries

To characterize the microbial community in human stool, 16S rDNA gene clonal libraries of gut bacteria were constructed using the sequences amplified by the universal primer sets 27F and 1492R for 16S rDNA.

One sample from each group was randomly selected for sequencing: a healthy adult on a vegetarian diet, a 44-year-old female (sample code B1-10); a healthy adult on a non-vegetarian diet, a 25-year-old male (B2-02); an IBD patient, a 57-year-old male (D1-09); and a colon carcinoma patient, a 42-year-old male (D2-03).

#### Sequencing of clonal libraries

The sequenced clones from the four stool samples, from each group of participants, belonged to the following phyla of the domain Bacteria: Proteobacteria, Bacteroidetes, Firmicutes, Actinobacteria, and Verrucomicrobiota.

**Figure 1.**
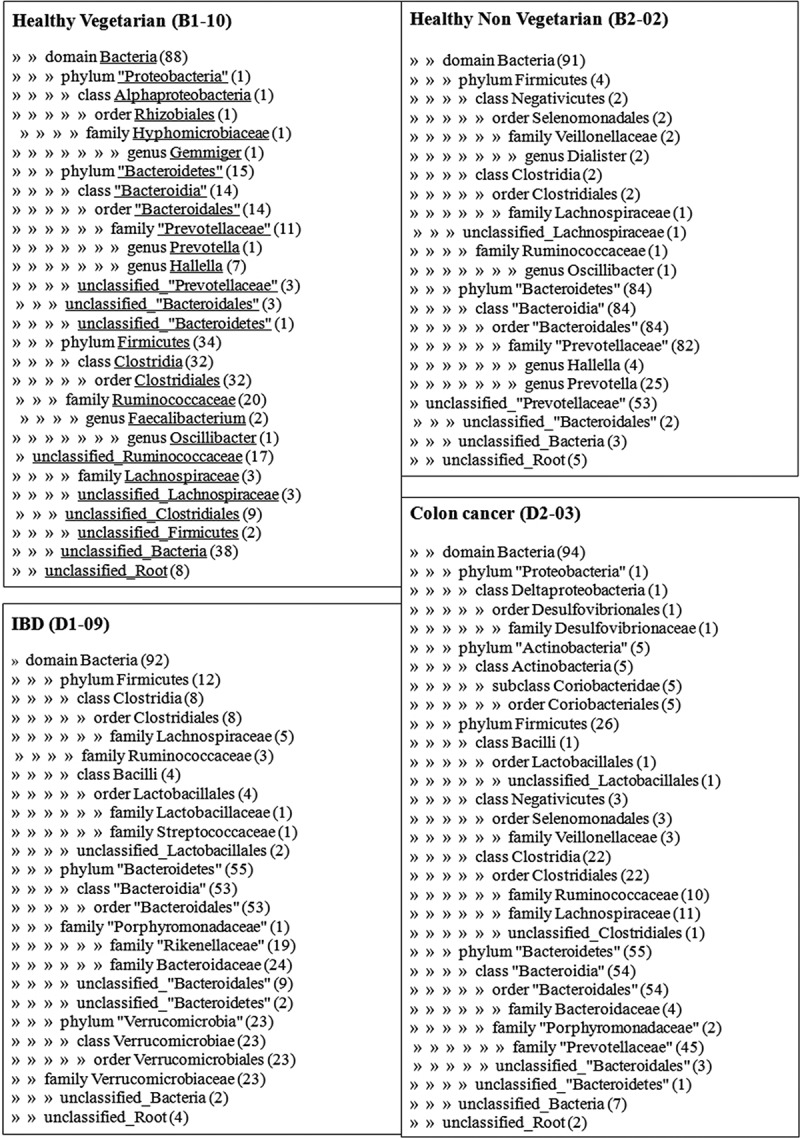
Ribosomal Database Project analysis of the gut microflora through sequencing of 16S rDNA clonal libraries in a healthy subject, a patient with inflammatory bowel disease (IBD), and a patient with colon cancer.

**Figure 2.**
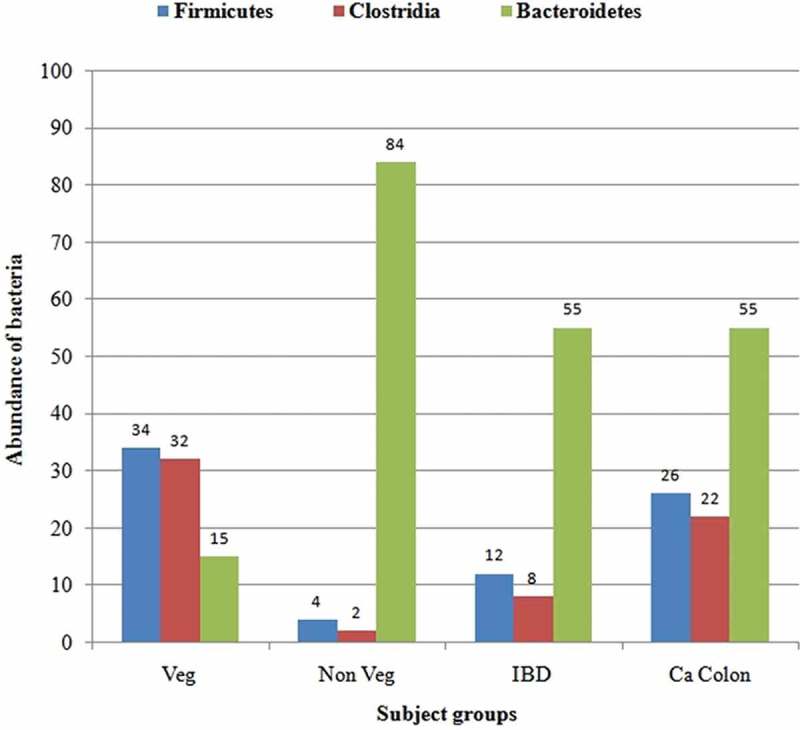
Abundance of major phyla in healthy subjects (Veg, vegetarian; Non Veg, non-vegetarian), a patient with inflammatory bowel disease (IBD), and a patient with colon cancer (Ca Colon).

The dominant phylum from the clones of the sample from the healthy vegetarian (B1-10) was Firmicutes (38%), followed by Bacteroidetes (17%). The phylum of 43% of bacterial clones from this group remained unclassified. In the sample from the healthy non-vegetarian (B2-02), the major phylum was Bacteroidetes (92%), followed by Firmicutes (5%). Clones from the sample from the IBD patient (D1-09) fell primarily into the phylum Bacteroidetes (60%), followed by Verrucomicrobiota (25%) and Firmicutes (13%). The most common bacterial phylum from the sample from the colon carcinoma patient (D2-03) was Bacteroidetes (59%), followed by Firmicutes (28%), and Actinobacteria (5%) (Figure 1). Abundance of major phyla in different groups is depicted in Figure 2.

Further analysis of sequences from Firmicutes revealed from samples from the healthy vegetarian (B1-10) were restricted primarily to the class Clostridia (95%), the predominant family being Ruminococcaceae. In the sample from the healthy non-vegetarian (B2-02), Phylum Firmicutes belonged to class Clostridia (50%) and class Negativicutes (50%). Firmicutes from the IBD patient (D1-09) were grouped into two classes, i.e. Clostridia (67%) and Bacilli (33%), and those from the colon cancer patient (D2-03) into three classes, i.e. Clostridia (85%), Negativicutes (12%), and Bacilli (3%). Sequences from Bacteroidetes from all four samples belonged to the class Bacteroidia, the predominant family being Prevotellaceae, which was 79% in the healthy vegetarian, 98% in the healthy non-vegetarian, and 83% in the colon cancer patient. The most common families of class Bacteroidia from the IBD patient were Bacteroidaceae (54%) and Rikenellaceae (35%). Sequences of the phyla Verrucomicrobiota and Actinobacteria were detected only in samples from the IBD patient (D1-09) and colon carcinoma patient (D2-03), respectively. Within the class Bacilli, bacteria belonging to the order Lactobacillales were detected from clones of the sample from the IBD patient (4%) and the colon cancer patient (1%). Within the class Clostridia, individual BLAST matches were mainly to the genera *Clostridium*, *Ruminococcus, Eubacterium*, and *Roseburia* (Figure 1).

## Discussion

The present study aimed to assess gut microbial diversity in healthy Indian adults and patients with colon carcinoma and IBD using a metagenomic approach. Studies of signature microbial diversity and bacteria–host interactions have been undertaken worldwide to demonstrate the role of commensal gut flora in the pathogenesis of diseases such as IBD and colorectal carcinoma. Although integrated metagenomic and metabolomics approaches have helped researchers to unfold the pattern of microbial flora in these groups of disorders, no data regarding the colonic microbial diversity in these subsets of the population are available from India.

### Vegetarian versus non-vegetarian adults

Sequencing of 16S rDNA from stool revealed Bacteroidetes and Firmicutes to be the predominant phyla in all four groups in our study. In the healthy adult control on a vegetarian diet, Firmicutes outnumbered Bacteroidetes. However, studies have shown that the phylum Bacteroidetes is dominant over Firmicutes in subjects on a strict vegetarian diet [31,32]. De Fillippo et al. reported that consumption of a diet restricted only to cereals, legumes, and non-animal proteins increases the proportion of Bacteroidetes over Firmicutes in vegetarians [31]. A decrease in the proportion of Firmicutes to Bacteroidetes has also been observed in vegetarians on a low-calorie diet [32,33]. Indian vegetarians usually consume a high-calorie diet containing dairy products and milk in addition to plant polysaccharides, which in combination probably contributed to the overrepresentation of Firmicutes compared to Bacteroidetes. Since milk products are rich in fat content, the dominance of the family Ruminococcaceae was also found in this group. An increase in the ratio of Firmicutes to Bacteroidetes in both human and mice models on a high-fat diet is well established [34]. In the non-vegetarian subject in our study, a relative abundance of Bacteroidetes over Firmicutes was observed. Within the phylum Bacteroidetes, the family Pervotellaceae harbored almost all the sequences. A long-term animal protein-based diet, composed mainly of meat, fish, and eggs, along with low levels of plant polysaccharides, increases the abundance of bile-tolerant organisms such as Bacteroidetes and decreases the level of Firmicutes. A high animal-based proteinaceous diet justifies the dominance of the Prevotellaceae family in our non-vegetarian control subject [33,35].

### IBD patient versus healthy adult

Gut microbial dysbiosis has also been observed in IBD patients. In a study by Frank et al., mucosal biopsies taken from patients with Crohn’s disease and ulcerative colitis showed a reduced abundance of Firmicutes and Bacteroidetes and a concomitant increase in Proteobacteria and Actinobacteria, compared to non-IBD controls [36]. A reduction in the proportion of Bacteroidetes with respect to Firmicutes was also observed in our IBD patient, who was on a non-vegetarian diet, compared to the healthy non-vegetarian control, but we did not find an increase in Proteobacteria and Actinobacteria compared to the healthy non-vegetarian adult. We found an abundance of bacteria of the phylum Verrucomicrobiota in the stool sample from the IBD patient. The IBD patient whose stool sample was subjected to sequencing was in the phase of remission, which may explain the mucosal colonization by Verrucomicrobiota, which is a part of the normal gut flora and rarely increases during active phases of mucosal inflammation. A higher percentage of individuals from both healthy control groups harbored lactobacilli in their stool compared to the colon carcinoma and IBD groups, as detected by conventional culture. This reinforces the fact that a lack of lactobacilli is associated with increased inflammation of the gut mucosa, which is a key pathogenic factor in both IBD and colon carcinoma. Results of conventional microbial culture showed the presence of *Lactobacillus* spp. in most of the samples in the vegetarian and non-vegetarian groups, while no *Lactobacillus* was found in the colon cancer samples, and it was found in only one IBD sample. Pathogenic *Clostridium* spp. were found in three out of eight samples from colon cancer patients, and no pathogenic flora was detected in healthy controls by conventional culture methods.

The present study had some limitations. We picked up 96 clones from the genomic library of one randomly selected participant from each group for sequencing. Selecting a larger number of clones for sequencing would have been a better method of demonstrating microbial diversity in our group of patients. However, 16S rDNA sequencing of all clonal libraries is highly time consuming and costly in a resource-limited setting [37].

### Colon carcinoma patient versus healthy adult

Comparison of the microbiota between the healthy non-vegetarian adult male and the colon carcinoma patient, who was also on a non-vegetarian diet, showed that the stool sample from the colon cancer patient had a higher percentage of Bacteroidetes than Firmicutes, but a lower Bacteroidetes:Firmicutes ratio than that in healthy adults. An increase in the abundance of Firmicutes compared to Bacteroidetes on phylogenetic analysis of luminal microbiota has been observed in Chinese patients [38]. Within the phylum Firmicutes, class Clostridia were predominant, and within the Clostridia, individual BLAST matches were mainly to genera such as *Clostridium*, *Ruminococcus, Eubacterium*, and *Roseburia*. As in the study by Wei et al., where a unique finding was the higher genetic diversity of gut flora in Wistar rats with precancerous lesions [39], we also found additional phyla like Actinobacteria, an increase in bacteria belonging to classes such as Lachnospiraceae and Viellonellaceae, and the highest number of unique band patterns on ARDRA in a stool sample from a patient with colon carcinoma. A carcinoma-associated microbiota characterized by an increase in the diversity of *Clostridium* spp. has been reported previously [40].

In conclusion, Indian patients with colon carcinoma and IBD showed unique patterns of microbial diversity compared to data from Western countries. Even healthy controls had different signature gut microbiota. The reason is likely to be the Indian dietary practices, which are very different from Western diets. Studies involving metagenomic and metaproteomic approaches that include larger subsets of participants are required to look into the colonic microbial diversity in the Indian population.
